# Metronomic Chemotherapy

**DOI:** 10.3390/cancers13092236

**Published:** 2021-05-06

**Authors:** Marina Elena Cazzaniga, Nicoletta Cordani, Serena Capici, Viola Cogliati, Francesca Riva, Maria Grazia Cerrito

**Affiliations:** 1 School of Medicine and Surgery, University of Milano-Bicocca, 20900 Monza (MB), Italy; nicoletta.cordani@unimib.it; 2 Phase 1 Research Centre, ASST-Monza (MB), 20900 Monza, Italy; capici@asst-monza.it (S.C.); viola.cogliati.vc@gmail.com (V.C.); 3 Unit of Clinic Oncology, ASST-Monza (MB), 20900 Monza, Italy; f.riva@asst-monza.it

**Keywords:** metronomic chemotherapy, clinical trials, mechanism of action

## Abstract

**Simple Summary:**

The present article reviews the state of the art of metronomic chemotherapy use to treat the principal types of cancers, namely breast, non-small cell lung cancer and colorectal ones, and of the most recent progresses in understanding the underlying mechanisms of action. Areas of novelty, in terms of new regimens, new types of cancer suitable for Metronomic chemotherapy (mCHT) and the overview of current ongoing trials, along with a critical review of them, are also provided.

**Abstract:**

Metronomic chemotherapy treatment (mCHT) refers to the chronic administration of low doses chemotherapy that can sustain prolonged, and active plasma levels of drugs, producing favorable tolerability and it is a new promising therapeutic approach in solid and in hematologic tumors. mCHT has not only a direct effect on tumor cells, but also an action on cell microenvironment, by inhibiting tumor angiogenesis, or promoting immune response and for these reasons can be considered a multi-target therapy itself. Here we review the state of the art of mCHT use in some classical tumour types, such as breast and no small cell lung cancer (NSCLC), see what is new regarding most recent data in different cancer types, such as glioblastoma (GBL) and acute myeloid leukemia (AML), and new drugs with potential metronomic administration. Finally, a look at the strategic use of mCHT in the context of health emergencies, or in low –and middle-income countries (LMICs), where access to adequate healthcare is often not easy, is mandatory, as we always need to bear in in mind that equity in care must be a compulsory part of our medical work and research.

## 1. Introduction

Molecular targeted agents differ from traditional chemotherapy agents in terms of administration schedules, toxicity profile and finally anticancer activity [1]. mCHT was coined for the first as the frequent administration of conventional chemotherapy drugs at low doses with no prolonged drug-free breaks in 2000’s by Kerbel [1] and Hanahan [2] in two different articles. Direct antitumor effect is not the primary mechanism of action. mCHT mainly exerts indirect effects on tumor cells, especially on their microenvironment by tumor angiogenesis inhibition, it also stimulates immune response of the host against cancer and acts on stromal tissue [2]. However, preliminary results of mCHT in different cancer types, mainly breast and lung cancer, showed interesting and promising results: mCHT remained confined to palliative settings for a long period, as erroneously considered to be devoid of antitumor activity. The preferred agents to be used in a metronomic regimen are oral drugs, considering that they could potentially be administered for longer periods with respect to traditional chemotherapy.

Different metronomic drug concentrations and schedules can exert different actions, so far, they can be modulated according to the setting of use, cancer type and patients’ preferences.

In this context, the most studied drugs for mCHT are cyclophosphamide (CTX), methotrexate (MTX), capecitabine (CAPE) and oral vinorelbine (VNR) in breast cancer, CAPE in gastro-intestinal cancers and oral VNR in NSCLC. With the advent of new drugs, especially immune checkpoint inhibitors, and a deeper knowledge of peculiar mechanisms of action of mCHT, it is advisable that these regimens become more widely used in clinical practice.

## 2. Metronomic Chemotherapy: Pre-Clinical Data

The effects of using mCHT with a single drug regimen have shown modest efficacy, while it increases rapidly when used in combination with other drugs, i.e., cyclophosphamide (CTX). In mice bearing tumors (received from human breast cell lines) CTX administered continuously in the drinking water with an antiangiogenic drug showed significant antitumor efficacy, and could be potentially applicable to chronic treatment [3]. Several preclinical studies shed light on the beneficial effects of metronomic chemotherapy and pave the way to their clinical evaluation. In recent decades, many studies have been conducted to clarify the mechanisms of action and therapeutic efficacy of mCHT. In this review, we retraced the steps related to mCHT starting from preclinical studies. Initially, it was proposed that this therapeutic regimen acted only on actively proliferating cells, especially on the endothelial cells of the tumor vasculature. The formation of new vessels in the growing tumor is required for nutrients and oxygen and proliferation. Judah Folkman first proposed that induction of tumor angiogenesis was required for malignant progression and later isolated from tumors a factor inducing angiogenesis, the Vascular Epidermal Growth Factor (VEGF). Then, Folkman proposed that blocking VEGF synthesis could starve the tumor, known as a dormant tumor [4].

During tumor growth, an angiogenic switch activates the endothelial cells in the harmful tissue. Activated endothelial cells proliferate and migrate, generating new vascular branches that promote tumor development and metastases [2]. At the beginning of the twentieth century, Miller et al. [5] proposed that some drugs used in chemotherapy regimens could act directly on actively proliferating tumor cells, and indirectly on the formation of new vessels and that these effects are annulled during the suspension of treatment by various mechanisms. Starting from this consideration, Browder and colleagues suggested a strategy to support the anti-angiogenic effects of chemotherapy: a continued administration of drugs at doses below the maximum tolerated dose (MTD) without long pauses between cycles [6]. They showed that a continuous low dose of cyclophosphamide is more effective than the standard schedule in cultured breast cells which have acquired drug resistance. Klement et al. demonstrated that chronic administration of low-dose vinblastine with anti-vascular endothelial growth factor receptor-2 (VEGFR-2) antibodies resulted in tumor regression [7]. Afterwards, numerous studies showed that mCHT inhibited the proliferation and circulation of endothelial cells (CECs) and endothelial progenitor cells (CEPs) and reduced the differentiation of immature endothelial cells, modulating pro- and anti-angiogenic molecules [6,8,9,10,11]. mCHT can shift the balance between pro-and anti-angiogenic factors inducing the synthesis and release of anti-angiogenic factors, as shown by Bocci et al. in in vitro studies on endothelial cells treated with diverse anticancer agents in the mCHT schedule and in in vivo in mice treated with low daily doses of cyclophosphamide (CTX). They demonstrated that in endothelial cells exposed to low and prolonged doses of drugs, the synthesis of thrombospondin-1 (TSP-1) is induced and directly mediates growth arrest and apoptosis. They also showed in human tumor-bearing immunodeficient mice treated with low daily doses of CTX the inhibition of angiogenesis. In particular, they observed an increase in circulating TSP-1 and an inhibition of pro-angiogenic factors’ recruitment [12]. Later, Bocci and others confirmed that mCHT with different drugs inhibits angiogenesis and tumor progression by increasing the expression and production of TSP-1 in animals bearing different types of tumors. Besides TSP-1, also other endogenous angiogenesis inhibitors have been enhanced by mCHT: endostatin (fragment of collagen XVIII), the first endogenous inhibitor of angiogenesis discovered, and angiostatin (fragment of plasminogen), soluble VEGF receptors (sVEGFRs) and Pigment Epithelium-Derived Factor (PEDF) [13]. Contrarily, mCHT decreases pro-angiogenic factors, such as inducible hypoxia factor (HIF-1α) and vascular endothelial growth factor (VEGF), during or after the administration of mCHT. 

HIF-1α is a hypoxia-regulated transcription factor, and it is the key regulator of the multistep metastatic cascade in solid cancers [14]. Moreover, hypoxia generated in the microenvironment of the tumor stimulates the expression of VEGF and the VEGF receptor-1-2, in both normal and neoplastic cells through an increase in transcription of HIF-1α and its mRNA stabilization. Different data support the hypothesis that HIF-1α regulates the multistep metastatic cascade at different checkpoints [14]. Schito et al., in 2020, showed decreased HIF-1α levels after mCHT administration, correlated with attenuated hypoxia and HIF-1α -sensitive microvessel densities, independently of systemic lung perfusion [15]. Considering that HIF-1α activity was correlated with the previously observed benefit of mCHT in the EMT6/CDDP pre-clinical metastatic breast cancer model [16], these findings together validate previous in vitro results and indicate that mCHT can induce HIF-1 α levels even in non-hypoxic cancer cell lines, a phenomenon which can be correlated with cancer stem cell enrichment leading to therapy resistance, tumor recurrence and metastases [17]. Moreover, low-dose of topotecan has been shown to inhibit HIF-1α and reduce VEGF-a expression in ovarian cancer cells in vitro. Other chemotherapeutic agents tested in various tumor cell lines demonstrated that VEGF release is decreased after treatment. These results were confirmed in several in vivo models, i.e., it has been shown that 5-FU is able to decrease the level of VEGF in colon cancer, while gemcitabine can reduce the aforementioned pro-inflammatory molecules in the in vivo model of pancreatic cancer. These effects are even more powerful when agents are used in combination, as shown by Mainetti et al. [18] in an in vivo model of mammary adenocarcinoma treated with a combination of doxorubicin and cyclophosphamide: they observed a reduction of VEGF and an inhibition of tumor growth and metastases. Furthermore, the anti-angiogenic effects of mCHT also result in the induction of tumor dormancy, the ability of tumor cells to survive without proliferation.

Tumor dormancy is regulated by the immune system, with a dynamic balance between cell proliferation and death due to apoptosis (immunological dormancy), or insufficient blood supply (angiogenic dormancy). At least three different mechanisms regulate dormant cancer cells: (1) intrinsic dormancy: cells are in mitotic arrest and are therefore less responsive to chemotherapy; (2) immunosurveillance: cells are able to escape immune disruption; (3) extrinsic dormancy: the switch from angiogenic to not-angiogenic effect [19]. New findings by Natale et al. in 2018 [13] indicate that mCHT leads to tumor dormancy, guiding to a direct impact on cancer cell proliferation [20]. In an orthotopic breast cancer xenograft model which simulates late-stage metastasis, a strong suppressive effect of metronomic 5-FU + cyclophosphamide (CTX) disease emerged [21]. 

Accumulated evidence suggests that mCHT induces senescence, a state of the stable proliferative arrest of cells. Repeated administration of low-dose topotecan leads to DNA damage and induced senescence in both MYCN-amplified neuroblastoma cells and in vivo in neuroblastoma xenografts, together with tumor regression [22]. 

Another relevant mechanism of action of mCHT consists in the recovery of the immune response, which acts against cancer cells by inhibiting T regulatory cells, myeloid-derived suppressor cells and stimulating dendritic cells [16]. Both innate and adaptive immune systems have an important role in the development and control of cancer. In this context, the effect on regulatory T cells (Tregs) is very relevant for metronomic treatments. Tregs are CD4+ CD25+ lymphocytes, and the expression of Forkhead box P3 (Foxp3) defines their property that determines the development and function of Tregs. They can inhibit antigen-specific immune response, both in a cytokine-dependent and cell-contact-dependent manner [8]). Tregs can thus inhibit the anti-tumor immune response by suppressing the activity of both tumor-specific (CD8+ cytotoxic T lymphocytes and CD4+ T helper cells) and tumor unspecific effector cells (natural killer [NK] and NK T cells) [8]. Treg cells have been found in increased proportions in different aggressive human cancers, which may be correlated with tumor progression and lack of treatment response [23]. Hence, impairment of Treg activity by either specific blockade or depletion is a method of enhancing immune response against tumor-associated antigens [8]. Many studies (preclinical and clinical) have documented the effect of low dose CTX on Treg cells; metronomic CTX reduces the number of Treg cells, suppresses the function of the Treg cells and increases both lymphocyte proliferation and memory T cells [9]. Metronomic schedules of vinblastine, paclitaxel and etoposide promote dendritic cell maturation at no toxic concentrations [24]. Similarly, an in vitro study on melanoma cells showed that vinblastine stimulated host immunity through DC maturation and cancer cell death-inducing apoptosis [10]. Myeloid-derived suppressor cells (MDSCs) are characteristic of an immature state and can inhibit T cells. However, their accumulation is limiting checkpoint blockade in tumors [25]. Metronomic paclitaxel administration leads to CD4 and CD8 T cell infiltration increase into tumor tissues and a reduction in the number of immunosuppressive cells [26]. Moreover, Orecchioni et al. [27] evidenced that VNR promoted the generation and maturation of myeloid antigen-presenting cells (APCs). They showed better outcomes using VNR associated with CTX and PD-L1 antibodies in breast cancer and lymphoma mice models. Recently, Khan et al. corroborated PD-L1 increase after metronomic CTX administration in orthotopic murine breast cancer. Nevertheless, when combined with simultaneous PD-L1 antibody treatment, the CTX regimens no-showed increased advantage [28]. 

Wu et al. [29] demonstrated that an intermittent, every 6-day repeating medium-dose CTX schedule at 90–140 mg/kg per injection could activate and sustained innate and adaptive immune responses following an initial transient lymphopenia. In the glioma model, the consequence of this regimen called MEDIC (Medium Dose Intermittent Cyclophosphamide) is the Natural Killer cell (NK) immune stimulation and the tumor response due to CD8+ T cell-mediated. In breast cancer models, low-dose metronomic CTX has demonstrated to be a key partner for immunotherapy blocking CTLA-4 [30].

Another evidence indicating the advantage of using mCHT is that it targets cancer stem cells (CSCs). These cells are capable of self-renewal and are involved in tumor generation and metastatic processes. CSCs are resistant to chemotherapy and they increase following MTD treatment, promoting tumor recurrence [31,32]. Recently, in some preclinical studies, the CD44+/CD24− cell population, described as cancer stem cells, showed reduction after being treated with paclitaxel [33]. Similarly, other studies demonstrated that cancer stem cells’ potentiality decreased due to inhibition of angiogenesis and directly inhibiting CSCs. Kerbel et al. found that metronomic cyclophosphamide reduced sphere-forming C6 rat glioma CSCs compared to MTD [34]. Moreover, new data emerged showing that the number of CD133+/CD44+/CD24+ cells, CD133+ precursors were reduced in a pancreatic cancer xenograft model following metronomic cyclophosphamide treatment by increasing thrombospondin-1 [35].

mCHT induces both indirect anti-cancer effects by inhibiting angiogenesis and activating the immune system, and direct anti-cancer actions. Recent studies showed that the combination of vinorelbine plus 5-FU is able to inhibit TNBC cell growth under mCHT schedule while promoting apoptosis and autophagy [36,37]. Other authors have shown that metronomic chemo-endocrine therapy leads to a higher expression of autophagy-related proteins, beclin 1 and LC3, and apoptosis-related markers expression, TUNEL and M30, in HR-positive breast cancer [38] in comparison to standard therapy. 

In this scenario, mCHT could represent a valid alternative to MTD regimens. 

**Figure 1:**
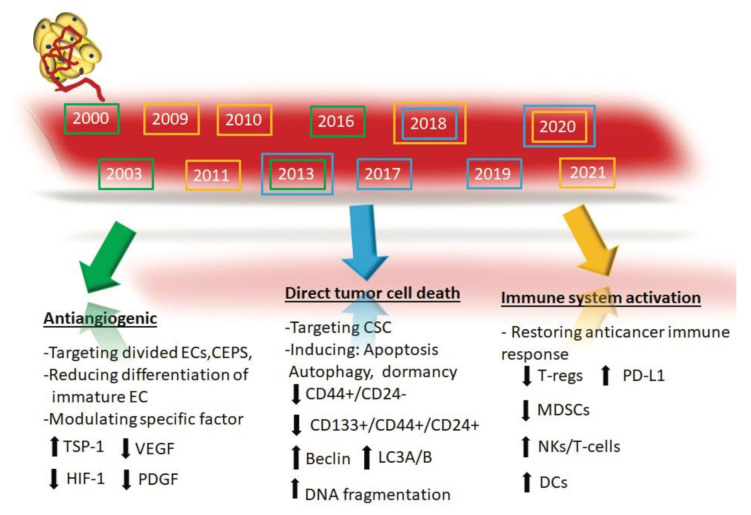
Timeline showing the discovery of the main impacts of the metronomic schedule. 1. Antiangiogenic: inhibiting the proliferating and the circulation of endothelial cells (EC) and by modulating the pro-and-antiangiogenic factors; 2. direct tumor cell death: targeting cancer stem cells (CSC) and inducing apoptosis, autophagy, and tumor dormancy; 3. activation of the anticancer immune response. These multiple mechanisms delineate metronomic as multitarget therapy.

Table 1 summarizes the possible major contributions to understanding the mechanisms of action of mCHT. Figure 1 represents a timeline showing the discovery of the main targets of the metronomic regimens. 

## 3. Metronomic Chemotherapy: Up to Date in Breast, Non-Small Cell and Colorectal Cancers

In recent years, several clinical trials on mCHT were conducted, demonstrating the growing popularity of this type of chemotherapy administration in different types of tumor. Six different meta-analyses have been published so far, mainly on breast cancer, [39,40] lung cancer [41,42] and glioblastoma [43].

### 3.1. Breast Cancer

Breast cancer is probably one of the most investigated tumors for mCHT. In patients with metastatic disease, the treatment is usually intended to improve the quality of life and reduce disease symptoms. mCHT provides an excellent alternative to conventional chemotherapy in terms of efficacy, with less side effects. Cyclophosphamide (CTX), methotrexate (MTX), vinorelbine (VNR) and capecitabine (CAPE) are the most frequent chemotherapeutic agents tested in metronomic trials.

The most extensively evaluated drugs as single agents are CAPE and VNR.

In 33 metastatic breast cancer patients, Taguchi and colleagues tested the efficacy of low dose of CAPE as first line treatment. Capecitabine was administered continuously according to the following schedule: 825 mg/m^2^ twice daily, for 21 out of 28 days. Median progression free survival (PFS) and overall survival (OS) were 6.9 and OS 24.8 months, respectively. This study demonstrated that CAPE was active and well tolerated in this schedule [23].

Another phase II study compared three different regimens in 323 metastatic breast cancer: CAPE 1000 mg/m^2^ twice daily for 14 of every 21 days, CAPE 650 mg/m^2^ twice daily regularly or classical CMF. Both the schedules of capecitabine were as effective as the traditionally administration of CMF, but with less side effects [9].

Fedele et al. evaluated the efficacy of continuous capecitabine at the dose of 1500 mg once a day in 60 pre-treated metastatic breast cancer patients. The study showed a clinical benefit rate (CBR) of 62%, a median time to progression (TTP) of 7 months and a median OS of 17 months [44].

VNR was tested in 34 elderly patients at the dose of 70 mg/m^2^ three times per week followed by one week off. This metronomic schedule was well tolerated (G3 neutropenia 6%) with a median PFS and an OS of 7.7 and 15.9 months, respectively [45].

De Iuliis and colleagues evaluated 30 mg of oral VNR every other day in 32 elderly patients. Clinical benefit rate was 50% and safety profile was optimal (no events grade 3 or 4) [46].

Some of the protocols also included the combined use with other chemotherapeutic agents, antiangiogenic, target therapy or endocrine therapy.

The combination of two oral agents, CTX and MTX (CM), given daily at low dose, was firstly studied among 63 patients, showing an overall objective response rate of 19% and an overall clinical benefit rate of 32% [47]. The same combination was analyzed in two other studies, confirming the low toxicity and effectiveness of the regimen [48].

Two phase I-II studies, VICTOR-1 and VICTOR-2, tested the metronomic administration of VNR and CAPE reporting percentages of CBR of 48–58% [49,50].

Different Phase II studies evaluated the metronomic administration of CTX and CAPE. The overall response rate was around 30–44%; Clinical benefit ranged from 53% to 57% [48,51].

Montagna et al. in another phase II trial tested the combination of triple drug oral chemotherapy. Metronomic regimen of VNR, CTX and CAPE (VEX regimen) in 25 previously untreated patients, showed a median TTP of 6.4 months [52].

VICTOR-6, the largest retrospective trial currently available, summarizes the use of mCHT in 584 patients with metastatic breast cancer. Indeed, significant results for VNR-based regimens were shown in first line setting (ORR around 36.7–44%; disease control rate around 82.4–88% [53].

Metronomic chemotherapy has been associated to antiangiogenetic agents, first of all Bevacizumab (BEVA) in order to improve the angiogenic action [54,55]. One of the best combinations was capecitabine (500 mg thrice daily), cyclophosphamide (50 mg daily) plus bevacizumab (10 mg/kg every 2 weeks) and showed a clinical benefit of 68%. One of the best combinations was capecitabine (500 mg thrice daily), cyclophosphamide (50 mg daily) plus bevacizumab (10 mg/kg every 2 weeks) and showed a clinical benefit of 68%.

In the phase III trial SAKK 24/09, mCHT with CAPE and CTX and BEVA was compared to paclitaxel and BEVA, as first line treatment, reaching a PFS of 8.5 months in metronomic arm, with no significant differences with respect to standard CHT [56]. 

Another phase II trial investigated the combination of CAPE, CTX and BEVA with erlotinib 100 mg daily, showing a CBR of 75% [57]. 

Finally, concerning the association with hormonal therapy, Schwartzberg et al. evaluated metronomic oral capecitabine and Fulvestrant in forty-one metastatic breast cancer. Median PFS and TTP were 14.98 and 26.94 months, respectively [58]. A further retrospective study tested the combination of oral metronomic CTX, MTX and Fulvestrant, resulting in prolonged CB (56%) [59].

In a recent meta-analysis of 22 clinical trials (around 1360 patients). promising results with mCHT in patients with advanced breast cancer were highlighted [60]. Several studies with mCHT have also been performed in the setting of early breast cancer, mainly in triple negative patients and as maintenance treatment after adjuvant treatment [61,62].

In a phase III study by Nasr et al., metronomic CM for 12 months after adjuvant therapy with carboplatin showed an increased OS [63].

Metronomic CAPE for one year, as maintenance therapy after standard curative treatment was studied in another recent phase III trial, SYSUCC-001, with evidence of significant improvement in DFS (83% vs. 73% at 5-years) [64].

Finally, several ongoing trials in different settings of breast cancer may provide further interesting data about mCHT. For instance, METEORA-II (NCT02954055) is comparing VEX-regimen to weekly paclitaxel in first or second line; MECCA trial (NCT02767661) is investigating the addition of an aromatase inhibitor to mCHT in hormonal positive breast cancer. 

### 3.2. NSCLC

Lung cancer is one of the most common cancer worldwide and causes about 20% of cancer deaths [65]. Despite of the advent of targeted- and immune-therapies, several lung cancer patients still receive chemotherapy during their Oncological disease.

Conventional chemotherapy may not be the best option for unfit or elderly patients due to its toxicity. This issue can be overcome by mCHT, an effective and well tolerated schedule for this subgroup of patients.

Several clinical trials investigated the role of mCHT in first-, second- and subsequent-line setting and its role as maintenance strategy in advanced NSCLC (NSCLC). In this clinical studies, different administration schedules were explored, starting from the use of single-drug treatment with vinorelbine [39,66] docetaxel, paclitaxel [67] or temozolomide [68] up to multiple-drug regimens (e.g., mVNR + cisplatin [69], platinum + oral etoposide [70]) and the combined use with antiangiogenic drugs (e.g., metronomic vinorelbine + sorafenib [71], cisplatin + metronomic oral VP16 + Bevacizumab [72], paclitaxel + gemcitabine + Bevacizumab [73], targeted drugs (e.g., oral vinorelbine + erlotinib) [74] or radiotherapy (e.g., metronomic cyclophosphamide + radiotherapy [75], dose-fractionated cisplatin + metronomic etoposide + radiotherapy [76], cisplatin + metronomic VNR + radiotherapy [77].

The most extensively studied drug for mCHT in NSCLC is oral vinorelbine (VNR) used as monotherapy, which has been evaluated in several phase II studies. The most studied schedule was a single agent use, three times per week, mainly patients considered unfit for platinum-based chemotherapy or already pretreated with different therapies.

In an individual patient-data meta-analysis on 418 subjects (80% of them having frailty characteristics) from 9 different studies, Pujol et al. reported a median PFS of 4.2 months and a median OS of 8.7 months with a 15.8% of grade 3–4 toxicity [41]. Another meta analysis on 509 patients from 11 clinical trials substantially confirmed these data, showing a median PFS of 3.46 months and an OS of 8.22 months with 16% of grade 3–4 toxicity [42].

Additional relevant information derived from the data of the most recent clinical trials can be added to these newsworthy meta-analyses.

A multicenter international retrospective analysis on 270 NSCLC patients treated in first, second or subsequent line with oral metronomic VNR at the dose of 50 mg, 40 mg or 30 mg thrice a week showed activity in terms of long-term disease stabilization with a RR of 17.8% and an overall DCR of 61.9% [66].

A randomized Phase II Tempo Lung trial, showed that in advanced NSCLC patients unfit for platinum-based first line chemotherapy, metronomic oral VNR at a fixed dose of 50 mg thrice a week significantly prolonged median PFS without grade 4 toxicity compared to standard schedule 60–80 mg/m^2^ [78].

A trial exploring the role of metronomic VNR as switch maintenance strategy after first-line platinum-based chemotherapy showed a prolonged PFS compared to best supportive care [79].

Therefore, metronomic oral VNR is nowadays a valid and safe option in some subgroups of patients with NSCLC. Future research should be focused to investigate its association with immune checkpoint inhibitors, a promising field given the immunomodulatory activity of metronomic chemotherapy [80].

### 3.3. Colorectal Cancer

Colorectal cancer (CRC) is one of the most common cancer and a leading cause of cancer death worldwide [65].

Fluoropyrimidines are the backbone of standard chemotherapy in colorectal cancer and had been the standard therapy for metastatic CRC (mCRC) until the approval of new chemotherapeutic agents (as irinotecan and oxaliplatin) and biological drugs (as bevacizumab, cetuximab and panitumumab). Recently regorafenib and TAS-102 were also approved as therapeutic options for heavily pretreated mCRC.

These treatments may be unsuitable for some frail elderly patients and for heavily pretreated patients that need a disease control with a low toxicity profile and good quality of life. Several studies demonstrate that mCHT is an effective and safe option for these subgroups of patients.

Some different types of drugs were evaluated as mCHT in CRC with promising results, such as CAPE [81,82,83,84], irinotecan [85] and CTX [65,86]. However, the most relevant data regard CAPE, an oral fluoropyrimidine.

Since 1998 continuous fixed daily dose of CAPE has been evaluated as a therapeutic option in CRC [81].

A retrospective analysis on 50 patients treated with continuous fixed dose of 1500 or 2000 mg daily with or without other therapies (e.g., irinotecan or fluorouracil) showed a low toxicity profile and none of the patients treated with metronomic CAPE as a single agent developed side effects of any grade [87].

Moreover, a more recent retrospective analysis on 68 pretreated or frail patients with mCRC administered with metronomic CAPE 1500 mg daily showed a disease control rate of 26% and a median OS of 8 months [88].

To this information, it is possible to add some case reports on mCHT reporting a good disease control with minimal side effects and a good quality of life in elderly or heavily pretreated patients [89].

Few clinical trials have also explored the role of metronomic CAPE as maintenance treatment.

A phase 3 randomized controlled trial has shown that maintenance therapy with metronomic CAPE plus bevacizumab following 6 cycles of conventional CAPE + oxaliplatin + bevacizumab significantly improved PFS compared to observation group (11.7 vs 8.5 months) without deterioration of quality of life [90,91].

The Nordic ACT2, a Phase III trial evaluated different therapies in the maintenance setting following first-line treatment in mCRC according to KRAS mutational status. Seventy-seven patients, selected according to KRAS mutational status were randomized to receive bevacizumab or metronomic CAPE (500 mg bid) without detecting differences in terms of PFS and OS [83].

The MOMA trial compared maintenance therapy with mCHT CAPE 500 mg/thrice per day and CTX 50 mg/die plus bevacizumab vs bevacizumab alone after 4 months of induction with FOLFOXIRI plus bevacizumab. Primary end-point (PFS) was not met in this trial [84].

Overall, these data support the role of metronomic CAPE as a therapeutic option in elderly, frail and heavily pretreated mCRC, while its role as maintenance therapy needs further investigations. Table 2 summarizes the most relevant results.

Table 2 summarizes the corner studies on breast, NSCLC and colon cancer.

**Table 1:** Summarizes the contributions of different Authors in understanding the mechanisms of action of mCHT.

Anti-Angiogenic Effect	Direct Tumor Cell Death	Immune System Activation
Browder, T. (2000) [6]	Vives, M (2013) [35]	Tanaka, H (2009) [24]
Klement, G. (2000) [7]	Kerbel, RS (2017) [34]	Tanaka, H (2009) [10]
Bocci, G. (2003) [12]	André, N (2017) [92]	Taguchi, T (2010) [23]
Mainetti, LE (2013) [18]	Cerrito, MG (2018) [37]	Stockler, MR (2011) [9]
Shaked, Y (2016) [16]	Ueno, T (2019) [38]	Orecchioni, S (2018) [27]
Schito, L (2020) [15]	Salem, AR (2020) [33]	Khan, KA (2020) [28]

**Table 2:** Summary of results of trials using mCHT in classical cancers (breast, lung, CRC).

Author (Year) Type of Cancer	Setting	Efficacy	Safety
	BREAST CANCER		
Garcia-Saenz et al. (2008) [56] Breast cancer	Phase II; Pretrated patients Pts 22 CTX 50 mg, MTX 1 mg/kg iv q14d Bevacizumab 10 mg/kg iv q14d Trastuzumab (in HER2 +)	CBR 63.6% PFS 7.5 m OS 13.6 m	Grade 3–4 Hypertension 4%
Dellapasqua et al. (2008) [55] Breast cancer	Phase II; Metastatic Pts 46 CTX 50 mg, Capecitabine 500 mg TID, Bevacizumab 10 mg/kg q14d	CBR 68% TTP 42w	Grade 3 Neutropenia 4% Hypertension 17%
Addeo et al. (2010) [46] Breast cancer	Phase II; I line Pts 34 VNR 70 mg/m^2^ thrice a week, 1-21 q28	ORR 38% PFS 7.7 m OS 15.9 m	Grade 3 Neutropenia 9% Anemia 9%
Taguchi et al. (2010) [23] Breast cancer	Phase II; I line Pts 33 CAPE 825 mg/m^2^ BID 1-21 q28	CBR 42% PFS 6.9 m OS 24.8 m	Grade 3 Neutropenia 6% HFS 15%
Stockler et al. (2011) [6] Breast cancer	Phase III; I line Pts 323 CAPE 1000 mg/m^2^ BID, 1-14 q21 vs. CAPE 650 mg/m^2^ BID continuously vs. classical CMF	OS 22 m CAPE groups vs. OS 18 m CMF group	Grade 3–4 21% vs. 35%
Fedele et al. (2012) [45] Breast cancer	Phase II; Pretreated patients Pts 58 CAPE 1500 mg daily continuously	CBR 62% TTP 7 m OS 17 m	Grade 3 HFS 5%
Yoshimoto et al. (2012) [52] Breast cancer	Phase II; I and II line Pts 45 CAPE 828 mg/m^2^ BID CTX 33 mg/m^2^ BID	ORR 44.4% PFS 12.3 m	Grade 3 Neutropenia 16%
Aurilio et al. (2012) [93] Breast cancer	Case-cohort report; Metastatic Pts 33 CTX 50 mg MTX 2.5 mg BID twice a week Fulvestrant 500 mg day 1, 250 mg day 1, 15, 28 q28d	CBR 56%	Grade 3 transaminases toxicity 3%
Wang et al. (2012) [49] Breast cancer	Phase II; Pretreated patients Pts 68 CTX 65 mg /m^2^ iv 1-14 q3w Capecitabine 1000 mg/m^2^ BID 1-14 q3w	ORR 30.3% CBR 53.0% TTP 5.2 m OS 16.9 m	Grade 3–4 5%
Schwartzberg et al. (2014) [59] Breast cancer	Phase II; Metastatic Pts 41 Capecitabine 750/1000 mg TID Fulvestrant 500 mg day 1, 250 mg day 1, 15, 28 q28d	TTP 26.9 m PFS 14.9 m OS 28.6 m	Grade 3 HFS 7,3%
Cazzaniga et al. (2016) [51] Breast cancer	Phase II; Metastatic Pts 80 VNR 20–40 mg daily, thrice a week and CAPE 500 mg TID continuously	CBR 48.8% TTP 7.5 m	Grade 3–4 Neutropenia 4% HFS 10%
De Iuliis et al. (2015) [47] Breast cancer	Phase II; Metastatic Pts 32 VNR 30 mg every other day continuously	CBR 50%	No Grade 3–4 events
	**LUNG CANCER**		
Hainsworth et al. (2001) [68] Lung Cancer	Phase II; I line Pts 39 Weekly Docetaxel 36 mg/m^2^	ORR 18% DCR 52% OS 5 m	Grade 3 Leukopenia 8% Anemia 13%
Correale et al. (2006) [71] Lung Cancer	Pilot Phase II; Stage IIIB/IV Pts 31 Weekly cisplatin 30 mg/m^2^ on days 1,8,14 and etoposide 50 mg/m^2^ on 21 of the 28 days	ORR 45.2% DCR 58.1% TTP 9 m OS 13 m	Grade 3 Anemia 33% Neutropenia 22.5%
Kouroussis et al. (2009) [69] Lung Cancer	Phase II; Pretreated patients Pts 31 Temozolomide 75 mg/m^2^ daily for 21 days every 28 days	ORR 6.5% DCR 16.5% TTP 2.4 m OS 3.3 m	Grade 4 Neutropenia 3.2% Thrombocytopenia 3.2%
Correale et al. (2011) [73] Lung Cancer	Phase II; I line Pts 45 Cisplatin (30 mg/m^2^ days 1–3), oral etoposide (50 mg days 1–15) and bevacizumab (5 mg/kg day 3) every 3 weeks	ORR 68.8% DCR 86.6% PFS 9.53 m	Grade 3–4 Mucositis 13.3% Pneumonia 17.8% Anemia 8.9% Leukopenia 8.9%
Noronha et al. (2013) [94] Lung Cancer	Retrospective Analysis; Pretreated and I line platinum-ineligible patients Pts 37 Weekly Paclitaxel 80 mg/m^2^	ORR 35% DCR 67.5% PFS 4 m OS 7 m	Grade 3 Anemia 8% Neutropenia 5.4% Sensory neuropathy 8%
Marquette et al. (2013) [74] Lung Cancer	Phase II; I line nonsquamous NSCLC Pts 33 Paclitaxel (80 mg/m^2^ days 1,8,15), gemcitabine (200–300 mg/m^2^ days 1,8,15) and bevacizumab (10 mg/m^2^ on days 1 and 15) for 6 cycles	PFS 9 m ORR 73% OS 30 m	Grade 3–4 Proteinuria 9% Pneumonitis 6% Fatigue 6%
Tan et al. (2015) [72] Lung Cancer	Phase I; ≥ II line Pts 48 Sorafenib 200 mg BID (starting dose) for 4 weeks with a fixed dose of VNR (thrice a week) at 60, 90 or 120 mg/week	ORR 8.9% DCR 66.7% PFS 4.4 m OS 8.2 m	Grade 3–4 HFS
Katsaounis et al. (2015) [70] Lung Cancer	Phase II; I line Pts 41 VNR 60 mg every other day and Cisplatin 80 mg/m^2^ in Cycles of 21 days	ORR 37.5% DCR 65.7% PFS 4.2 m OS 12 m	Grade 3–4 Neutropenia 31.4%
Revannasiddaiah et al. (2016) [76] Lung Cancer	Retrospective Analysis; Stage II and III Pts 139 CTX 50 mg daily and radiotherapy vs. radiotherapy alone	ORR 41.9% PFS 3.1 m (CTX + RT) vs. ORR 33.9% PFS 2.55 m (RT alone)	N/A
Sutiman et al. (2016) [75] Lung Cancer	Phase I (dose escalation); ≥ II line Pts 30 VNR 40 mg/m^2^ day 1,8 every 21 days (starting dose) and Erlotinib 100 mg daily (starting dose) mVNR 100 mg/week (day 1,3,5) (starting dose) and Erlotinib 100 mg daily (starting dose)	ORR 38% (conventional schedule) ORR 29% (metronomic schedule)	Grade 3–4 Neutropenia 13% Neutropenia 36%
Pastina et al. (2017) [77] Lung Cancer	Retrospective analysis; Metastatic Pts 69 Cisplatin 30 mg/m^2^ on days 1–3 plus oral etoposide 50 mg daily from day 1 to day 15 and bevacizumab 5 mg/kg on the day 3 every three weeks. Palliative RT allowed (45 pts)	OS CHT 12.1 m vs. OS CHT + RT 22.12 m	No significant adverse events or toxicity-related interruptions
Pujol et al. (2019) [42] Lung cancer	Meta-analysis; Advanced and metastatic Pts 418 VNR 30–50 mg thrice a week	OS 8.7 m PFS 4.2 m	Grade 3–4 15.8%
Platania et al. (2019) [80] Lung Cancer	Randomized Phase II; Maintenance Pts1 20 VNR 50 mg thrice a week vs. Best Supportive Care	PFS 4.3 m OS 11.8 m DCR 53.3% vs. PFS 2.8 m OS 14.2 m DCR 44.6%	Grade 3–4 Neutropenia 11%
Camerini et al. (2019) [67] Lung Cancer	Retrospective Analysis; Stage IIIB/IV Pts 270 VNR 30–50 mg thrice a week	ORR 17.8% DCR 61.9% TTP 5 m OS 9 m	Grade 3–4 2%
Xu et al. (2020) [43] Lung Cancer	Meta-analysis; Stage IIIB/IV and advanced NSCLC Pts 509 mVNR as single agent at different doses	ORR 12% DCR 48% PFS 3.46 m OS 8.22 m	Grade 3–4 16%
Camerini et al. (2021) [79] Lung Cancer	Randomized Phase II; Stage IIIB/IV Pts 167 VNR 50 mg thrice a week vs. VNR 60–80 mg/m^2^	G4PFS 4.0 m G4DCR 45% PFS 4.3 m DCR 63.9% OS 7.1 m vs. G4PFS 2.2 m G4DCR 26% PFS 3.9 m DCR 63.4% OS 7.6 m	Grade 3–4 25.3% vs. Grade 3–4 54.4%
Provencio et al. (2021) [78] Lung Cancer	Phase II; stage III Pts 55 Induction with 2 cycles Cisplatin 80 mg/m^2^ every 21 days plus VNR 50 mg thrice a week 🡪 Concomitant treatment with the same dose of cisplatin and VNR 30 mg/day	PFS 11.5 m ORR 66.2% OS 35.6 m	Grade 3–4 21.5% (during induction) 24.5% (during concomitant treatment)
	**COLORECTAL CANCER**		
Budman et al. (1998) [82] Solid Tumors (Colorectal Cancer)	Phase I; Solid Tumors unresponsive to standard therapy Pts 33 (17 CRC) CAPE 110 m/m^2^ (starting dose)	1 mixed response and 1 SD	Most frequent grade 3–4 toxicity at MTD: diarrhea
Herben et al. (1999) [95] Solid tumors (Colorectal Cancer)	Phase I; Solid Tumors refractory to standard therapy Pts 33 (CRC 22) Irinotecan 12.5 mg/m^2^/day for 14 days every 3 weeks (starting dose)	2 partial response (1 CRC)	Dose limiting toxicity: gastrointestinal events (diarrhea with or without nausea and/or vomiting)
Lokich (2004) [88] Solid Tumors (Colorectal Cancer)	Retrospective analysis; MetastaticPts 50 (26 CRC) CAPE 1000 mg or 2000 mg daily as single agent or in association with other therapies (irinotecan weekly, 5-FU infusion weekly, radiation therapy, other)	N/A	Grade 2–3 14% No toxicity for CAPE as single agent
Allegrini et al. (2008) [96] Colorectal Cancer	Pharmacokinetic and pharmacodynamic study; Metastatic pretreated Pts 20 3 levels of metronomic dose 1.4, 2.8, 4.2 mg/m^2^/day continuously	SD 20% PFS 2.07 m OS 8.4 m	No grade 3–4 events
Nannini et al. (2009) [90] Colorectal Cancer	Case Report; Elderly metastatic elderly (CRC and gastric cancer) Pts 3 (2 CRC) CAPE 1000 mg daily	SD all 3 pts	N/A
Allegrini et al. (2012) [87] Colorectal cancer	Phase II; metastatic pretreated Pts 38 CTX 500 mg/m^2^ ev day 1 m from day 2 50 mg p.o. once daily plus UFT 100 mg twice a day plus celecoxib 200 mg twice a day	SD 45% PFS 2.7 m OS 7.1 m	No grade 3–4 events
Ogata et al. (2013) [86] Colorectal Cancer	Phase II; First line Pts 45 Irinotecan 60 mg/m^2^ on days 1,8,15 plus S-1 80 mg/m^2^/day on days 3 to 7, days 10 to 14 and days 17 to 21 every 4 weeks	ORR 48.9% PFS 8.1 m OS 20.9 m	Grade 3–4 Neutropenia 8.9% Anemia 4.4% Anorexia 6.7% Diarrhea 6.7%
Romiti et al. (2015) [89] Colorectal Cancer	Retrospective analysis; Metastatic pretreated or frail Pts 68 CAPE 1500 mg daily continuously	DCR 26% OS 8 m	Grade 3 HFS 2.9% Anemia 1.5% Diarrhea 1.5%
Simkens et al. (2015) [92] Colorectal Cancer	Phase III; Maintenance Pts 558 CAPE 625 mg/m^2^ twice daily continuously plus bevacizumab 7.5 mg/kg every 3 weeks vs. Observation	PFS 11.7 m OS 21.6 m vs. PFS 8.5 m OS 18.1 m	Grade 3–4 HFS 23% vs. Grade 3–4 HFS 0%
Hagman et al. (2016) [84] Colorectal Cancer	Phase III; Maintenance Pts 77 CAPE 500 mg twice daily vs. Bevacizumab 7.5 mg/kg	PFS rate at 3 m 66.7% PFS 3.7 m OS 28 m vs. PFS rate at 3 m 75% PFS 3.9 m OS 26.4 m	Grade 3–4 15.2% vs. Grade 3–4 20.6%
Cremolini et al. (2019) [85] Colorectal Cancer	Ranzomized phase II; Maintenance Pts 232 CAPE 500 mg thrice daily plus CTX 50 mg daily plus bevacizumab 7.5 mg/kg vs. Bevacizumab 7.5 mg/kg	PFS 10.3 m OS 22.5 m vs. PFS 9.4 m OS 28 m	Grade 3–4 HFS 9.1% vs. Grade 3–4 HFS 0%

## 4. Metronomic Chemotherapy: Areas of Novelty

Considering that until 2010 clinical data on mCHT was limited to small series of patients on retrospective analyses and in occasional case reports, we first performed a literature search using 2 different databases (PubMed, Metacrawler) with the aim of identifying the clinical trials published between 2010 and 2021 by using the keyword metronomic chemotherapy. Then, we refined our search, excluding trials regarding breast, non-small cell lung cancer and colorectal cancers, on which there are lots of reviews recently published, and to which we give only the above short comment regarding the state of the art. While we want to focus and present here after 3 different areas of novelty:

1. Novel combinations and novel cancer types
2. New areas of research (innovative methods to monitor response to mCHT, new biomarkers already applicable in the clinical practice)
3. How the COVID-19 pandemic (and potentially other health emergencies) has changed the position of mCHT in clinicians’ strategies.

### 4.1. Novel Cancer Types, Novel Regimens

#### 4.1.1. Head and Neck Cancer

Palliative systemic therapy plays an important role in recurrent, relapsed, or newly diagnosed head and neck cancers that are not amenable to any localized therapy upfront [97]. In low and middle-income Countries (LMICs), the unavailability of regimens for palliation in patients with head and neck cancer remains a big social and ethical problem, because of their cost. In a phase I-II study [98] Patil et al. recruited the first 15 patients, identifying 9 mg/m^2^ of MTX as the optimal biologic dose (OBD). In the Phase II part of the same study, further 91 patients were recruited: the 3-month PFS rate was 71.1% (95% CI, 60.5% to 79.3%), the 6-month OS rate was 61.2% (95% CI, 49.2% to 67.8%), and the ORR was 42.9% (95% CI, 33.2% to 53.1%; *n* = 39). In a subsequent randomized Phase 3 trial, the same Authors [99] enrolled adult patients aged 18–70 years, already candidate to receive palliative systemic therapy for relapsed, recurrent, or newly diagnosed squamous cell carcinoma of head and neck (SCHNC), to either oral mCHT (MTX 15 mg/m^2^ once per week + Celecoxib 200 mg twice per day, or Cisplatin 75 mg/m^2^ once every 3 weeks for six cycles). Patients treated with mCHT had better outcomes in comparison with those who received intravenous cisplatin, even if this latter is the standard of care in LMICs. Median OS was 7.5 months (IQR 4.6–12.6) in the mCHT group compared with 6.1 months (3.2–9.6) in the cisplatin group. Grade 3 or higher toxicity found in the group treated with mCHT was 19% and 30% in the standard-of-care one (*p* = 0.01). Even if some critical issues can be raised about these results, this study is a clear example of how to balance the possibility of offering treatment to patients in LMICs, without reducing the effectiveness of the treatment too much.

- At the moment there are no ongoing trials on this topic. Confirmatory studies are strongly warranted.

#### 4.1.2. Glioblastoma

In glioblastoma (GBL), metronomic administration of temozolomide (TMZ) at the dose of 50 mg/m^2^/day has emerged in the last 10 years as a potential option of rescue treatment for recurrent tumors. Three Phase 1 trials have been published [100,101,102], mainly investigating the role of mCHT in combination with anti-angiogenic agents such as bevacizumab, sorafenib and bortezomib, with the idea of exploiting the well-known angiogenesis inhibition mechanism of mCHT.

- Ongoing trials: none for the time being. 1 trial, which investigated the combination of surgery + Gliadel^®^ wafer implantation + Limited Field Radiation Therapy with concomitant daily temozolomide at the dose of 75 mg/m^2^ followed by monthly temozolomide given at the same dose (75 mg/m^2^/day for 21 days monthly), has been recently completed. No results are yet available.

#### 4.1.3. Ovarian Cancer

Therapies for recurrent ovarian cancer are still of limited clinical benefit and have a deep impact on the patient’s quality of life. New strategies, which also take into consideration the quality of life of patients, are urgently needed.

No randomized Phase 3 trials are available at the moment using mCHT [103]. Different Phase 2, even randomized, studies [104,105] and some Phase 1 trials [106] have been published. These trials mainly investigated the role of mCHT as maintenance therapy after MTD induction chemotherapy, or the potential use as a true second-line treatment. Different agents have been studied, being oral CTX and Topotecan the most studied drugs. Similarly, the two most investigated combinations of mCHT are with anti-angiogenic compounds, or tyrosine kinase inhibitors (TKIs).

- Ongoing studies: NCT01175772 is a study that investigates the efficacy of a novel mCHT regimen in patients with ovarian cancer, in the setting of maintenance treatment after a response induction by the conventional MTD treatment which consists of Carboplatin and Paclitaxel. The regimen studied includes CTX combined with two agents which can act as indirect angiogenic inhibitors: (a) celecoxib, a selective COX-2 inhibitor and (b) low-dose MTX, as a suppressor of the inflammatory cascade. The study is based on the hypothesis that the combination of the drugs, administered orally and continuously for one year, are able to suppress the process of recovery of residual disease, with the purpose of prolonging the TTP, and possibly the OS.

Well-designed, powered, and randomized studies are strongly awaited to answer the numerous questions still open in this cancer type, especially the role of mCHT as maintenance therapy.

#### 4.1.4. Hematologic Malignancies/Non-Lymphoma

Acute myeloid leukemia (AML) in older adults is different in terms of both biology and clinic and different compared to when it occurs in younger patients. It is characterized by adverse chromosomal abnormalities, stronger intrinsic resistance, and lower tolerance to chemotherapy. In patients aged more than 60, cure rates are under 10% despite intensive chemotherapy, and most of them have an adverse prognosis within the first year. In this setting of care, mCHT has emerged as a potential therapy to control both advanced and refractory disease. Three trials have been reported till now: a pilot prospective study in 32 AML patients aged more than 60 years and not suitable for curative treatment, were treated with daily oral 6-mercaptopurine 75 mg/m^2^ [107]. The median OS was 6 months. Males showed better prognosis in comparison to women: median OS was 7 months (95% CI: 5.4–8.6) versus 3 months (95% CI: 1.5–4.4; *p* = 0.008). Toxicity was mild with no Grade 4 toxicities and no episode of febrile neutropenia.

A multi-center controlled trial randomized unfit AML patients to receive either mCHT (Etoposide 50 mg/m^2^ for 5 days plus 6-Mercaptopurine (6MP) 60 mg/m^2^ for 2 weeks and prednisolone 40 mg/m^2^ for 2 weeks) or an appropriate dosage of oral hydroxyurea to maintain the number of white blood cell counts to less than 10,000 cell/mm^3^. The OS rate was higher in the group receiving mCHT in comparison with the group receiving palliative treatment at both 6 (HR 0.60; 95%CI 0.36, 1.02; *p*-value 0.060) and 12 months (HR 0.66; 95%CI 0.41, 1.08; *p*-value 0.097) with borderline significance [108].

- No studies are ongoing nowadays. Even if we are aware that lots of strategies are available for this hematologic cancer, we still suggest to further explore what has only been sketched in these preliminary studies, especially for frail patients

#### 4.1.5. Pediatric Cancers

Progresses in childhood cancer treatment, in the last decades, resulted in a survival rate of over 80% in high-income countries, whereas the survival rate among children with cancer still remains very poor in LMICs. Options of treatment are usually limited, especially when tumor progression appears after 1–2 lines of standard chemotherapy. mCHT has been explored in this setting of care with conflicting results.

Two different areas of research can be identified:

1. mCHT as maintenance treatment in different pediatric cancer [109]
2. Metronomic combination of different chemotherapy drugs [110,111]
3. mCHT in combination with other drugs [112,113,114]

##### Metronomic Chemotherapy as Maintenance Treatment

Due to its continuous schedule and multimodal way of action, mCHT is the ideal treatment to consider as maintenance strategy after induction standard chemotherapy.

A landmark study evaluated mCHT with oral MTX and CTX (MC) continuously in patients with high-grade operable osteosarcomas (OSTs) of the extremities [115]. After neoadjuvant chemotherapy with MTX, adriamycin, and platinum (MAP), followed by surgery, patients were randomized to receive further 31 weeks of MAP or 73 weeks of MC after MAP. At 5 years, the Event-Free-Survival (EFS) rates were 61% in the MAP-MC group and were 64% the MAP-alone group, respectively, without any statistically significant difference. These data on the current EFS results seem not to support the clinical practice use of metronomic MC after standard chemotherapy for no metastatic OST, however, they should not preclude further studies in the maintenance setting for these patients.

##### Metronomic Combination Chemotherapy

Metronomic combination of multiple drugs has been explored in different settings, especially relapsed, refractory childhood tumors.

Minturn et al. [111] investigated the role of metronomic Topotecan in a population of 26 young patients with different brain tumors. Topotecan was administered orally at the dose of 0.8 mg/m^2^/day for 21 consecutive days followed by a 1-week stop. Objective response was observed in only 2 patients, but this strategy could be evaluated in larger trials to confirm this partial activity.

One of the most important trials in this setting has been recently published by a group historically working on this topic [116]. Authors randomized children with pediatric extracranial solid tumors that showed progression after at least 2 lines of chemotherapy to mCHT or placebo. The study consisted of a 4-drug oral metronomic regimen of daily celecoxib and thalidomide with alternating periods of etoposide and cyclophosphamide versus placebo. Median age of the 108 out of 123 enrolled young patients was 15 years; after a median follow-up of 2.9 months, PD was observed in 100% of the patients in the placebo group vs 96.4% in the mCHT group (*p* = 0.24). However, median PFS and OS were similar in the two groups (PFS: HR = 0.69; 95% CI, 0.47–1.03, *p* = 0.07; OS: HR, 0.74; 95% CI, 0.50–1.09, *p* = 0.13). In a post-hoc subgroup analysis, patients receiving more than 3 cycles and those without a bone sarcoma appeared to derive benefit from mCHT.

##### Metronomic Chemotherapy in Combination with Modulating Agents

Metronomic administration of various agents from different classes, with different mechanisms of action can induce the inhibition of angiogenesis and promote immunostimulatory effects as well as apoptotic activities. The continuous administration of drugs such as cyclophosphamide, methotrexate, etoposide and more recently vinorelbine and capecitabine, can exert a cytotoxic effect on both circulating endothelial cells and their progenitors, while no effect on leukocytes. Their combination with other drugs, usually used for different indications, has been investigated in different trials, the two most promising seem to be sirolimus and celecoxib.

Qayed et al. [112] explored the role of sirolimus in a Phase 1 trial in combination with celecoxib BID, and alternating etoposide and CTX administered every 3 weeks in 18 patients younger than 30 years of age with recurrent, refractory, or high-risk solid and brain tumors. The study established the recommended dose for sirolimus at 2 mg/m^2^.

Another study [117] investigated the combination of sirolimus with oral metronomic topotecan and CTX in children with relapsed or refractory solid tumors. Out of 21 patients enrolled into the study, 4 showed a prolonged stable disease whereas the biomarker correlative study demonstrated a modulation of angiogenic pathways with this combination.

Together with sirolimus, celecoxib, an anti-COX2 agent, was widely investigated in combination with mCHT.

Berthold et al. [118] compared a fourth-drug regimen containing etoposide, CTX, vinblastine and celecoxib for up to 24 months in relapsed/refractory high grade neuroblastoma, comparing outcome to 274 matched patients using different variables: the curves for second-event free and overall survival demonstrated no differences between mCHT regimen and standard dose-intensive chemotherapy, with a very low toxicity, especially in terms of leukopenia and thrombocytopenia. In another trial, Robinson et al. evaluated the combination of celecoxib with thalidomide, and fenofibrate, with alternating 21-day cycles of low-dose cyclophosphamide and etoposide in 97 children with recurrent or progressive cancer, showing an ORR of 13.1% and a DCR of 50.5%. Analysis of the correlative biomarker study showed that baseline serum thrombospondin levels were significantly higher in responding patients than in those who progressed (*p* = 0.009).

The METRO-MALI-02 trial [113] reported the preliminary efficacy and safety of the combination of metronomic vincristine/cyclophosphamide/methotrexate/valproic acid in 7 children with refractory cancer, mainly neuroblastoma, showing 2 partial responses. This trial is of particular interest as it introduces the new concept of “metronomics” that is the combination of mCHT and drug repositioning: in LMICs this could be a strategy to offer affordable targeted therapies, even if larger trials are needed to clarify definitive results.

mCHT in association with radiotherapy was one way to explore the role of mCHT as a sensitizer agent. These trials are also supported by preclinical data: Pasquier et al. demonstrated that a combination of beta-blockers and vinblastine-based metronomic chemotherapy in Ras-transformed vascular endothelial cells in vitro and tumor spheroid 3D model induced the induction of cell death apoptosis. They also reported the beneficial effect in the treatment of advanced angiosarcoma [119].

Sharp et al. [114] investigated the association of metronomic TMZ combined with standard radiotherapy with the aim of boosting up the anti-angiogenic activity of TMZ. Fifteen children with diffuse intrinsic brainstem glioma received TMZ at the dose of 85 mg/m2/day for 6 weeks, followed by metronomic TMZ monotherapy at the same dose, as maintenance therapy. Six-months and 1-year OS rates were 80% and 20% respectively. The most common toxicities were prolonged lymphopenia and thrombocytopenia.

Ongoing trials: there is 1 recruiting trial at the moment (NCT02446431) in pediatric leukemia patients, which evaluates the role of metronomic schedule in patients who are in remission after completion of front-line therapy.

Table 3 summarizes the most relevant results.

### 4.2. Novelties in Imaging Techniques and Biomarkers

#### 4.2.1. Imaging Techniques

One of the principal difficulties in evaluating the response to mCHT is that its peculiar mechanisms of action often does not evidence a tumor shrinkage as other MTD therapies do, a phenomenon also reported for more recent treatments, such as immune check-points inhibitors.

Some Authors recently proposed diffusion MRI (dMRI) and dynamic contrast-enhanced (DCE) MRI for the evaluation of treatment response to mCHT in the 4T1 mammary tumor model of locally advanced breast cancer [121]. Their results suggest that dMRI and DCE-MRI should be potential biomarkers for assessing the tumor response to mCHT. This study also hypothesizes that MRI, together with the derived pathology observations of administering mCHT with 5-FU induces changes in the tumor vasculature, so far demonstrating an antiangiogenic effect which adds to the cytotoxic effect already demonstrated by different studies.

#### 4.2.2. Biomarkers and Beyond

For a long time, one of the main limits of mCHT use in clinic was the absence of predictive biomarkers. Considering the multitarget action of mCHT, biomarkers for angiogenesis inhibition, such as trombospondin-1, circulating VEGF and CEC, biomarkers expression of immune system enhancement, like TAM and TILs, and tumor burden have been explored in different studies.

##### Biomarkers of Angiogenesis

Different biomarkers of angiogenesis, like Vascular Endothelial Growth Factor (VEGF), Vascular Endothelial Growth Factor Receptors (VEGFRs), Circulating Endothelial Cells (CECs) and its progenitor cells, as well as thrombospondin-1 (THBS-1), hypoxia-inducible factor-1α (HIF-1α) have been extensively, even though not systematically studied, showing conflicting results.

In a Phase I study, evaluating the combination of dalteparin, CTX MTX and daily prednisone as therapy for metastatic breast cancer, Wong et al. [122] evaluated vascular endothelial growth factor (VEGF) and soluble vascular endothelial growth factor receptor (sVEGFR) levels as markers of response, finding any correlation with response. Similar results were reported by Lansiaux et al. [123] for thrombospondin-1, another biomarker for angiogenesis inhibition, in a Phase II study. Other Authors [124] evaluated different predictive/prognostic factors, namely HER2, Ki-67, thymidine phosphorylase (TP), VEGF and VEGFR expression in the tumor samples of 62 patients with metastatic breast cancer treated with oral CTX and MTX: only, TP was found to be associated with PFS.

An interesting study by Mayer et al. [125], evaluating the safety and tolerability of vandetanib and CM metronomic combination in 23 heavily pretreated breast cancer patients, reported that proteomic analyses showed changes in platelet content of angiogenesis regulators (VEGFR and platelet factor 4), suggesting that platelet proteome may serve as a pharmacodynamic marker of angiogenesis inhibition.

Similar suggestions regarding the potential role of CEC and their changes during treatment were reported by Buckstein et al. [126] in a trial evaluating the efficacy, safety and anti-angiogenic effects of melphalan plus lenalidomide in chronic myeloid leukemia: Authors reported that transient spikes in CECs were described in responder patients.

As described in the studies provided as examples, correlations between markers of angiogenesis and trials objectives are rare and variable and unfortunately negatively contributed to the use of mCHT in the clinical practice.

##### Biomarkers of Immune System Enhancement

Research on biomarkers outside the angiogenesis field may be more promising.

One of the most recent findings suggested that high levels of tumor-infiltrating lymphocytes (TILs) are associated with improved patients’ outcomes in both triple negative and HER2+ve breast cancer, whereas the role of TILs in Luminal cancer remains unknown. Montagna et al. [127] investigated the relationship between TILs and TTP in 92 metastatic Luminal breast cancer patients treated with the VEX metronomic combination. Authors showed that high TILs levels are significantly associated with a worse TTP in patients treated by metronomic chemotherapy.

In a different setting, [128] other Authors evaluated the combination of anti-PD-L1 and mCHT in advanced sarcomas; in the correlative study, they found that a strong infiltration of macrophages, most of which expressing the inhibitory enzyme indoleamine 2,3-dioxygenase 1 (IDO1), thus suggesting a potential correlation between immunosuppressive tumor microenvironment, which finally could produce macrophage infiltration and IDO1 pathway activation.

##### Mutations and Tumor Burden

One of the most robust results in the field of correlative studies evaluating the role of mutations and tumor burden was recently reported by Munzone et al. [129]: authors investigated the prognostic and predictive value of a selected number of somatic mutations in TNBC enrolled in IBCSG Trial 22-00, finding any correlation between the most common mutations occurring in TNBC, like PIK3CA, BRAF, KIT and AKT1, and recurrence.

The characterization of the gut microbiota of cancer patients under different regimens and drugs may describe a new role of gut microbiota associated with drug efficacy. Guan et al. [130] evaluated the composition and the function of gut microbiome associated with metronomic CAPE schedule compared to conventional dosage of the same drug. In this context, the fecal samples of HER2-ve advanced breast cancer patients treated with metronomic CAPE as maintenance treatment were collected and analyzed by 16S ribosome RNA gene sequencing.

This study suggested that the prevalent gut microbial composition of Slackia (9.2 vs. 32.7 months, *p* = 0.004) vs. Blautia obeum can impact on outcome (median PFS: 32.7 vs. 12.9 months, *p* = 0.013) at univariate and multivariate analyses. The gut microbiota of patients receiving mCHT should therefore be different in terms of diversity, composition, and function from those who are receiving conventional chemotherapy, and the presence of specific bacterial species may act as microbial markers associated with drug resistance and finally prognosis.

## 5. Metronomic Chemotherapy, Public Health Emergencies and Equality in Treatment Access: The Lesson of COVID-19 Pandemic

COVID 19 pandemic is definitively the biggest emergency National health services have ever faced in modern times.

During the pandemic, healthcare systems of all Countries were promptly reorganized to deal with the severe crisis of acute patients arriving in hospital emergency rooms: this has had a great impact on the management of cancer patients.

As stated by different position papers published during this period of pandemic, oral chemotherapy was recommended to limit the access of cancer patients to hospitals and to guarantee the continuation of treatment in some cases [131]. COVID-19 pandemic forced us to review our strategies, in order to maintain the clinical care, and the consent process [116]. The acceptable and demonstrated efficacy, together with the low toxicity of oral mCHT, makes it a reasonable and potential option during health emergencies but not only. Considering that the affordable problem of subscribing new drugs, and guaranteeing equitable access to care for all, still remains a big problem and will become one of the most important topics that we, as oncologists, have to face in the upcoming years.

## 6. Discussion

Metronomic chemotherapy in treating cancer has started quietly, confined to later lines of therapy, in an almost exclusively palliative setting.

Starting from the early twenties, the use of mCHT has become more and more widespread, breast cancer serving as the forerunner, but very rapidly followed by other types of cancer, especially lung cancer and pediatric tumors.

The cornerstone principles of mCHT are (1) the easy-to-use way of administration, (2) the low incidence of side effects, and (3) the multi-modal mechanisms of action. All these factors have significantly contributed to the common use of mCHT in several other types of cancer (head & neck, ovarian, AML, GBM).

mCHT is now recommended in ABC-ESMO guidelines for metastatic breast cancer and adopted in different national guidelines, also in those countries, like Vietnam, where the routine use of this strategy is very recent.

In October 2017, a group of 10 International Experts in the management of breast cancer, with extensive experience in cancer treatment, convened to develop an expert report aimed at describing the current status of the use of mCHT for the treatment of advanced breast cancer, based on literature evidence and KoL opinion [132].

A full consensus was reached concerning the acknowledgement that mCHT is not simply a different way of administering chemotherapy but a truly new treatment option. Independently of HR status, mCHT could be an advantageous option for elderly patients, who are often under-treated simply because of their age. The experts strongly suggest that the ideal patients for mCHT are those with hormone receptor (HR)-positive tumors or those with triple-negative disease.

The best-known effect of mCHT is on angiogenesis inhibition, but exciting new data are on the way regarding potential activity on immune system activation.

Different preclinical data suggest the potential combination of mCHT with anti-angiogenic drugs (i.e., bevacizumab, sunitinib, erlotinib), or with anti-PD-L1 agents and some clinical data are also available, even if showing conflicting results [56,103] and apparently no added benefit for mCHT. We strongly believe that these combinations should deserve further investigations, in both preclinical and clinical settings: a better understanding of the complex crosstalk activated or inhibited by the combinations of these agents is essential to fully explore the role of mCHT.

Regarding breast cancer, some areas are already been consolidated: real-life studies have suggested us that mCHT is an effective treatment especially in HR+/HER2-ve advanced breast cancer patients without visceral crisis and with limited tumor burden, and elderly patients [132]. Some areas of interest for clinical purposes still remain open, with limited data available till now, especially: (1) maintenance treatment in high-risk triple-negative metastatic patients; (2) extended adjuvant treatment in early TNBC patients, radically resected, at high risk of relapse.

Regarding NSCLC, in our opinion that data are well consolidated in unfit advanced patients [78], for whom mCHT sometimes can represent the only option of treatment, considering that more aggressive regimens and combinations with immune check inhibitors are precluded.

Regarding colorectal cancer, the ideal use of mCHT is probably the maintenance setting [84], even if different areas, such as elderly or unfit patients, still deserve further evaluation.

The important results showed in different studies in pediatric tumors also represent a big step forward, especially for those children living in LMICs for whom every new drug can do the difference between cure and palliative treatment.

Despite promising preliminary results showed in different studies, mainly with oral mCHT-based regimens, only limited data has been available for a long time regarding the right dose of the different drugs to be used in the metronomic administration.

Unfortunately, well-designed and powered studies, with robust exploratory biomarker endpoints, are still lacking. We all know that these trials are usually very expensive and need large cooperation among centers.

It is our opinion that the absence of important pharmaceutical companies’ support, providing funding for basic research, and new trials design could compromise future research in mCHT. For these reasons, we strongly believe that academic research should make up for this deficiency.

Finally, most of the recent literature contributions are arriving from South-East Asia, South Africa and Latin America, as well as LMICs, rather than Northern world Countries, where mCHT was discovered in its beginnings. This is, in our opinion, an important warning that some areas of the world still have the need for effective, easily accessible low-cost drugs, and that it is our moral duty to contribute to its development.

## 7. Conclusions

Metronomic chemotherapy has long been confined to the palliative setting: now that the amount of pre-clinical and clinical data has become so important, it’s time to move to different areas, in particular to early onset of breast and colon cancer, to special populations of NSCLC patients, and to consolidate its use in pediatric cancers. Oral schedules, which can be administered at home, together with the very favorable toxicity profile, could be of great help in treating patients in low-resource countries.

## Figures and Tables

**Table 3:** Summary of results of trials using mCHT in different cancers (H&N, GBL, ovarian cancer, AML).

Author (Year) Type of Cancer	Setting	Efficacy	Safety
HEAD AND NECK CANCER			
Patil (2019) [94] Oral cancer	Phase I-II Platinum-resistant Erlotinib 150 mg (fixed dose) orally once per day, celecoxib 200 mg (fixed dose) orally twice per day, oral MTX 9 mg/m^2^	3-months PFS rate 71.1% 6-months PFS rate 61.2% ORR 42.9%	Grade 3–5 Hyponatremia 16.4% ALT increase 5.5% AST increase 4.1% Thrombocytopenia 4.1% Neutropenia 4.1% Anemia 4.1%
Patil (2020) [95] H&N cancer	Phase III First-line MTX 15 mg/m^2^ methotrexate once per week + Celecoxib 200 mg twice per day or CDDP 75 mg/m^2^ once every 3 weeks	OS 7.5 vs. 6.1 months 6-months OS rate 62.4% vs. 51.12%	Grade 3–5 Hyponatremia 13% Neutropenia 4% Anemia 2%
**BRAIN TUMORS**			
Reynés (2014) [98] GBM	Phase I First-line TMZ 50 mg/m^2^, three daily doses IRI (MTD 100 mg/m^2^ days 8, 22 every 28 days)	ORR 8.3% SD	Grade 3–4 Hematologic 40% Non-hematologic 30%
Peters (2018) [96] GBM	Phase I/II Heavily pre-treated VOR 200 or 400 mg po alternating 7 days on then 7 days off Bevacizumab 10 mg/kg iv every 2 weeks TMZ 50 mg/m^2^ po daily	6-months PFS rate 53.8% 6-months OS rate 84.6% ORR (RANO) 43.6%	Grade 3–4 Hematology 7 pts Non-hematology 15 pts
**OVARIAN CANCER**			
Gupta (2019) [120] Recurrent epithelial ovarian, Fallopian tube Primary peritoneal cancer	Phase II, randomized 52 patients CTX 50 mg or CTX 50 mg + Celecoxib 400 mg × 2	ORR 4% vs. 4% OS 9.69 vs. 12.5 months	Grade 3–4 Fatigue 8% vs. 23% Lymphoenia 4% vs. 19% Anemia 15% (Arm B)
Hall (2020) [100] Ovarian Fallopian tube Primary peritoneal cancer	Phase II, randomized 117 patients Heavily pretreated CTX 100 mg Nintenanib 200 mg (150 mg) daily Or Placebo	PFS 2.9 vs. 2.6 monthsOS 6.8 vs. 6.4 months	Grade 3–4 64% vs. 54%
Zsiros (2021) [99] Ovarian	Phase II 40 patients Platinum resistant/sensitive Pembrolizumab 200 mg Bevacizumab 15 mg/kg every 3 weeks CTX 50 mg once daily during a 21-day treatment cycle	ORR 47.5% PFS 10 months	Grade 3–4 Lymphopenia 7.5% Hypertension 15%
**HEMATOLOGIC MALIGNANCIES**			
Kapoor (2016) [103] AML	Phase II Elderly 32 patients 6-mercaptopurine 75 mg/m^2^	OS 6 months	No Grade 4
Pongudom (2020) [104] AML	Phase III Unfit, 81 patients 50 mg per m^2^ of Etoposide 50 mg/m^2^ × 5 days + 6 MP 60 mg/m^2^ × 2 weeks + Prednisolone 40 mg/m^2^ × 2 weeks Or appropriate dosage of oral hydroxyurea to maintain WBC <number of white blood cell 10,000 cell/mm^3^	OS 4.03 vs. 2.3 months 6-months OS rate 24.2% vs. 17.7%	Grade 3–4 Diarrhea 0.02% vs. 0.03% Bleeding 0.11% vs. 0.05% Febrile neutropenia 0.85% vs. 0.62% Systemic infection 0.78% vs. 0.69%
